# Efficacy of different spinal nerve roots for neuromodulation of micturition reflex in rats

**DOI:** 10.18632/oncotarget.23950

**Published:** 2018-01-04

**Authors:** Jianshu Ni, Xiaohu Wang, Nailong Cao, Jiemin Si, Baojun Gu

**Affiliations:** ^1^ Department of Urology, Shanghai Jiao Tong University Affiliated Sixth People’s Hospital, Shanghai, China

**Keywords:** spinal nerve root, micturition reflex, ventral root, dorsal root, pseudorabies virus

## Abstract

Electrical stimulation of peripheral nerves controlling the bladder is an alternative, nondestructive medical treatment for urinary incontinence and retention. In this study, we aimed to identify the most efficient sensory and motor spinal nerve roots involved in the micturition reflex. Unilateral L5-S2 dorsal and ventral roots were electrically stimulated, and bladder reflex contractions were recorded under isovolumetric conditions. Repeated stimulation of the L6 and S1 dorsal roots not only abolished bladder reflex contractions but also induced a poststimulation inhibitory effect, whereas repeated stimulation of the L5 and S2 dorsal roots had no effect. Only the L6 ventral root directly caused bladder contraction when ventral roots L5-S2 were stimulated in sequence. Upon retrograde tracing using pseudorabies virus (PRV), the sacral parasympathetic nucleus of the L6 segment had more PRV-positive cells than the other segments, though the S1 segment of the dorsal root ganglia had the highest density of PRV-positive neurons. These results suggest the L6 ventral root is most efficient in producing detrusor muscle contraction, and the S1 dorsal root best inhibits the micturition reflex.

## INTRODUCTION

Electrical stimulation of the sacral spinal nerve is an established therapy for patients with urge incontinence, increased frequency, and urinary retention after failure of first-line pharmacotherapy and was approved by the U.S. Food and Drug Administration in the 1990s [1, 2]. Although sacral neuromodulation has been used for over two decades, its exact effect on the micturition reflex and its mechanism for improving urinary incontinence and urinary retention have not been completely delineated.

Over the past few decades, studies have focused on the effect of neuromodulation on experimentally induced voiding dysfunction via electrical stimulation of the spinal nerves. In one study, electrical stimulation of different frequencies and intensities was delivered at the L6 spinal nerve, and a frequency of 10 Hz and intensity of 0.6 mA were found to maximally inhibit the volume-induced bladder reflex contraction (BRC) [3]. It has also been reported that, in a particular time window after complete spinal transection, electrical stimulation (20 Hz, 1.5-4 V) of the S1 spinal root appeared to inhibit reflex bladder activities [4, 5]. In another study, bladder activation was observed when electrical stimulation passed through the nerve cuff with electrodes placed on the extradural S2 spinal roots [6]. Although these studies demonstrated the effect of spinal nerve electrical stimulation on bladder function, the stimulation parameters used and the results obtained varied. Moreover, few studies accurately identified which spinal nerves most affected bladder function or how ventral roots (VRTs) and dorsal roots (DRTs) affect micturition in rats. Several studies demonstrated that electrical stimulation of the L6-S3 VRTs at a range of frequencies and intensities had no or minimal impact on bladder contraction [7–9], which is inconsistent with the positive results demonstrated using sacral anterior root stimulation found by Brindley [10].

In an attempt to eliminate the discrepancies caused by different stimulation parameters and varied animal models, we aimed to establish a convenient rat model with parameter-fixed stimulation for demonstrating and comparing the effects of neuromodulation on reflex bladder activity. We also sought to identify the superiority of different spinal roots involved in micturition.

## RESULTS

### Effect of DRT stimulation on BRC

**Figure 1:**
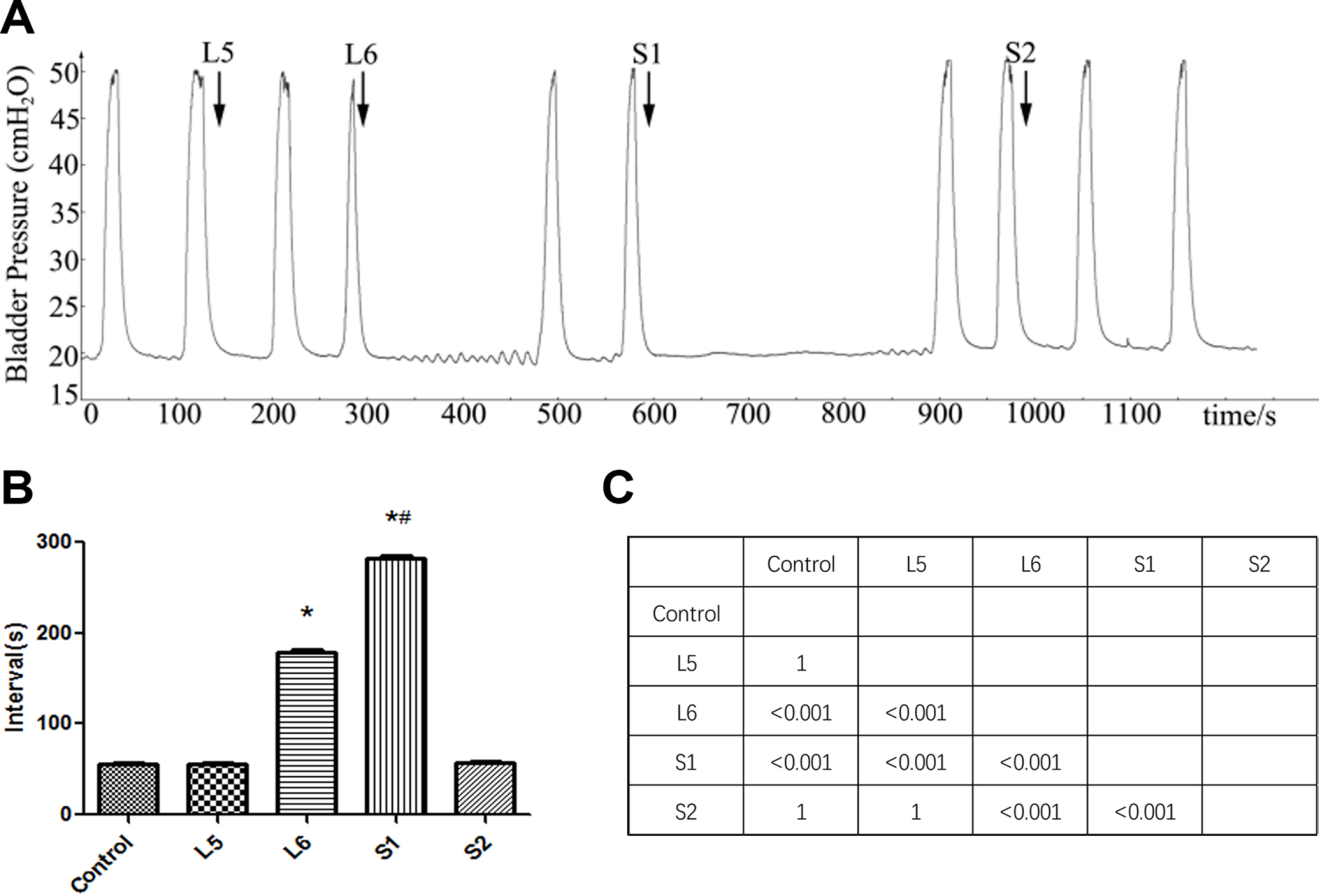
Effect of DRT stimulation on bladder reflex contraction (BRC) (**A**) L5-S2 DRTs were electrically stimulated in sequence. Repeated stimulation of L6 and S1 DRTs not only abolished BRC but also induced a poststimulation inhibitory effect, whereas electrical stimulation of L5 and S2 DRTs had no effect. The arrow shows the stimulation. (**B**) Interval of BRC during DRT stimulation. The BRC intervals during L6 and S1 DRT stimulation were longer than in the control (^*^P < 0.01). S1 DRT stimulation resulted in significantly stronger inhibition than L6 DRT stimulation (^#^*P* < 0.01). (**C**) The matrix presents the *P* values.

There was no significant change in BRCs during electrical stimulation of L5 and S2 DRTs. However, repeated stimulation of L6 and S1 DRTs at the same parameters not only abolished BRCs but also induced a poststimulation inhibitory effect (Figure 1A). The BRC intervals during L6 and S1 DRT stimulation were longer than those in the controls (*P* < 0.01). S1 DRT stimulation resulted in significantly stronger inhibition than L6 DRT stimulation (*P* < 0.01) (Figure 1B).

### Effect of VRT stimulation on BRC

**Figure 2:**
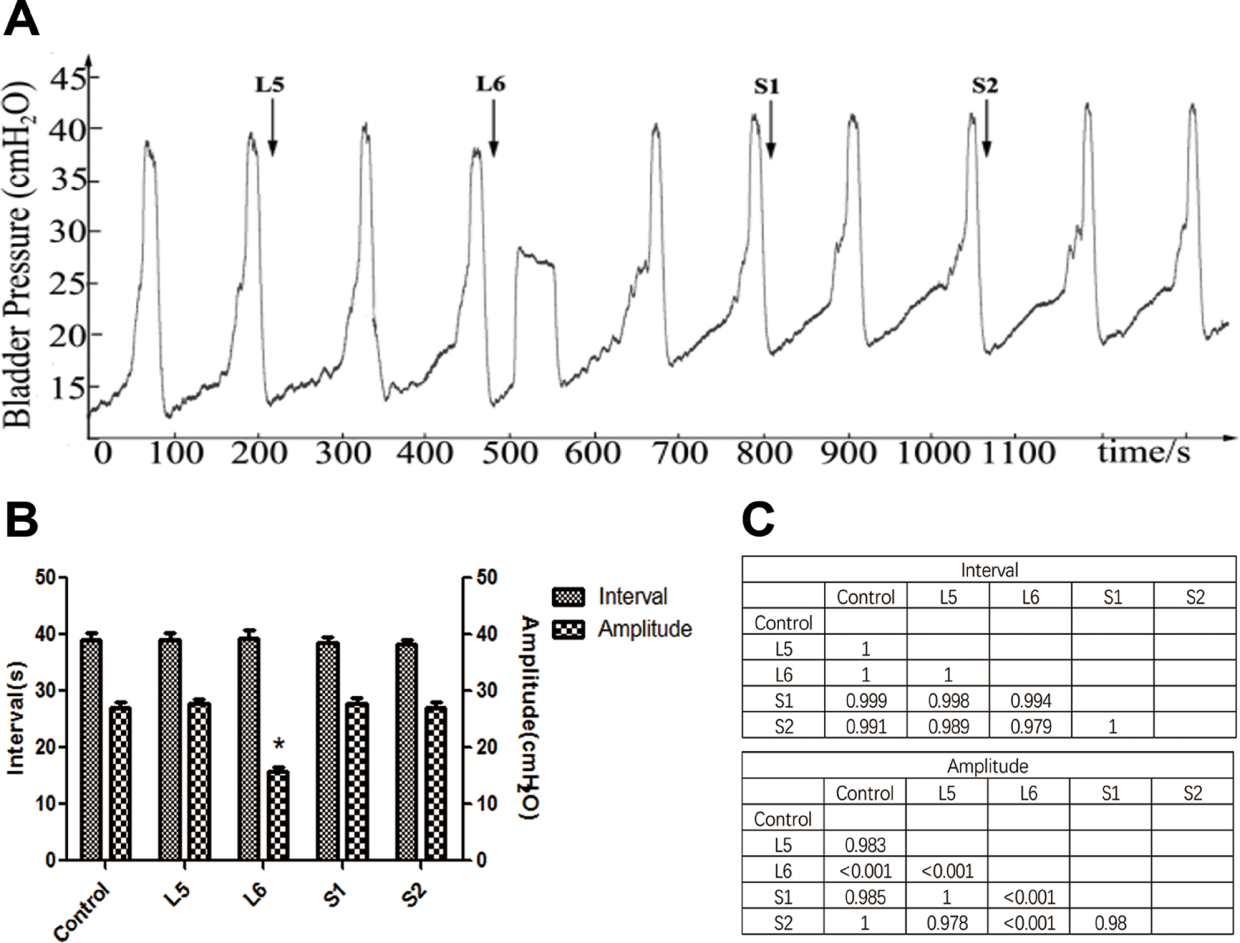
Effect of VRT stimulation on bladder reflex contraction (BRC) (**A**) L5-S2 VRTs were electrically stimulated in sequence. Repeated stimulation of L6 VRT caused a poststimulation bladder contraction, whereas electrical stimulation of the L5, S1, and S2 VRTs had no effect. The arrow shows the stimulation. (**B**) Interval and amplitude of BRCs during VRT stimulation. The amplitude of induced BRCs during L6 VRT stimulation was lower than in the control (^*^P < 0.01), whereas the interval showed no significant difference. (**C**) The matrix presents the *P* values.

There was no significant change in BRCs (interval or amplitude) during electrical stimulation of L5, S1, and S2 DRTs. Repeated stimulation of the L6 VRT at the same parameters caused a poststimulation bladder contraction (Figure 2A). The amplitude of the stimulated contraction was lower than in the control (*P* < 0.01), whereas there was no significant difference in the interval (Figure 2B).

### Pseudorabies virus (PRV) retrograde tracing

**Figure 3:**
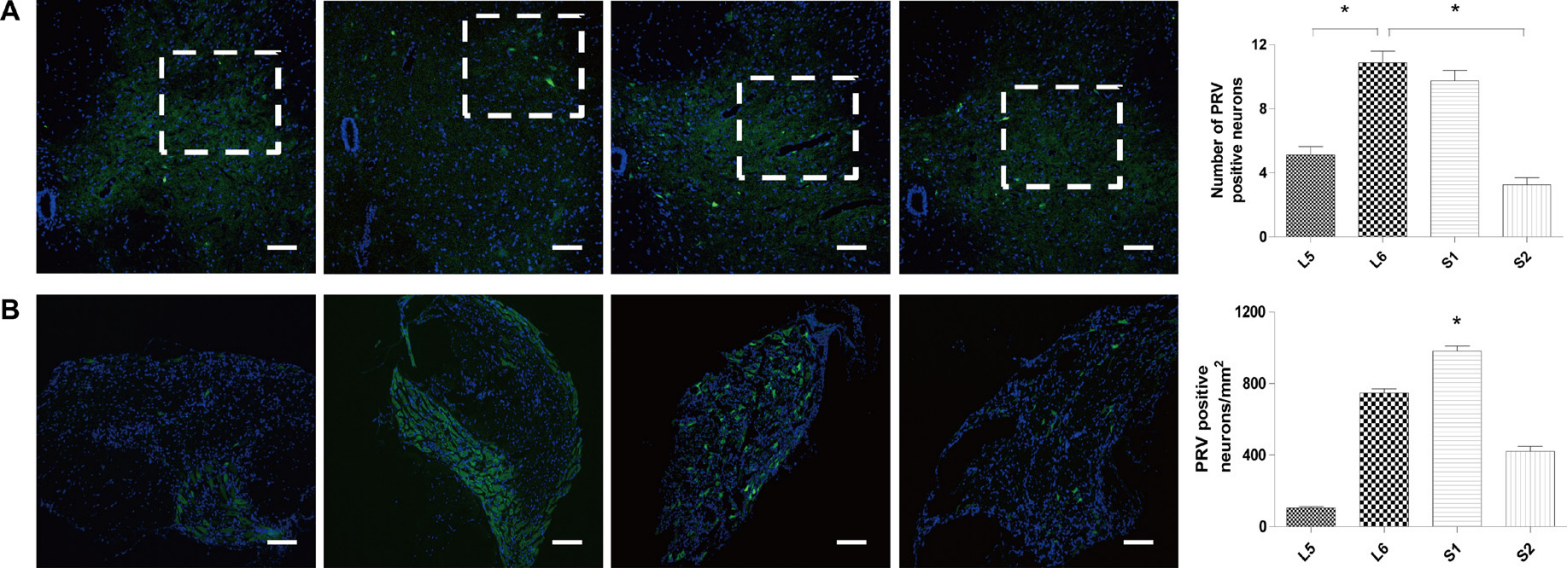
Results of PRV retrograde tracing (**A**) PRV-positive neurons in the SPN of the L5, L6, S1, and S2 segments. The most PRV-immunoreactive cells were found in the SPN of the L6 spinal segment, followed by the S1, L5, and S2 segments. (**B**) PRV-positive neurons in L5, L6, S1, and S2 DRGs. The S1 DRG was found to have the highest density of PRV-positive neurons, followed by the L6, S2, and L5 DRG. (Scale bar = 50 μm, ^*^P < 0.05.).

PRV-positive neurons were found in L5 to S2 spinal cord segments and dorsal root ganglions (DRGs). In spinal cord, the sacral parasympathetic nucleus (SPN) of the L6 spinal segment had the most PRV-immunoreactive cells, followed by the S1, L5, and S2 segments (Figure 3A). In DRGs, the S1 segment was found to have the highest density of PRV-positive neurons, followed by the L6, S2, and L5 segments (Figure 3B).

## DISCUSSION

This study was performed to establish a convenient animal model for analyzing the effects of neuromodulation on micturition reflex and to identify the superiority of different spinal nerve roots involved in micturition. We used fixed stimulation parameters, and the protocols were determined according to previous studies. Ren et al. [7] used a frequency of 10 Hz and a pulse width of 0.1 millisecond but a different current intensity. They found that 200-μA electrical stimulation of the dorsal spinal nerve root most strongly inhibited the isovolumetric bladder contraction. Fu et al. [11] demonstrated the effect of electrical stimulation of the L5-S2 ventral spinal root on defecation reflex. They used a monophase square wave, intensity of 2 mA, pulse duration of 0.2 milliseconds, stimulation frequency of 5 Hz, and persistent period of 5 seconds. Su et al. [3] tested the effects of 10 minutes of electrical stimulation of different spinal roots on isovolumetric bladder contraction and found that 10 Hz most strongly inhibited bladder contraction. Thus, we chose the parameters of 10 Hz, 0.1 millisecond, and 0.2 mA. In addition, because the average isovolumetric bladder contraction interval is 30 seconds, we chose continuous stimulation for 30 seconds to demonstrate whether stimulation would completely inhibit the contraction.

Our results showed that repeated stimulation of L6 and S1 DRTs at the given parameters not only abolished BRCs but also induced a poststimulation inhibitory effect, whereas electrical stimulation of the L5 and S2 DRTs had no effect. S1 DRT stimulation inhibited BRC significantly longer than L6 DRT stimulation. Interestingly, at our current parameters, we found that stimulation of the L6 VRT caused a poststimulation bladder contraction, whereas stimulation of other VRTs had no effect. However, the amplitude of the induced contraction was only half that of the control, and the contraction interval demonstrated no significant difference. PRV-positive neurons were found in L5 to S2 spinal nerves. In spinal cord, the most PRV-immunoreactive cells were found in the SPN of the L6 spinal segment, followed by the S1, L5, and S2 segments. In DRGs, the S1 segment was found to have the highest density of PRV-positive neurons, followed by the L6, S2, and L5 segments.

Neural control of micturition is a complex process involving both somatic and autonomic pathways participating in a fine-tuned integration of segmental lumbosacral reflexes [12]. Although a number of studies were designed to determine the mechanism underlying sacral neuromodulation, it remains complicated and uncertain [13–15]. Regarding the neurophysiologic control of micturition, the therapeutic options for neuromodulation of bladder dysfunction are clear: (1) activate the motor fibers innervating the detrusor muscle to produce bladder contraction, increase intravesical pressure, and promote bladder emptying; and (2) activate the sensory fibers to suppress detrusor instability and detrusor hyperreflexia to increase functional bladder capacity and prevent incontinence [16].

Lumbosacral ventral and dorsal nerve roots, located intradurally in the cauda equina, include mainly efferent and afferent axons, respectively. In this study, we found that DRT stimulation could induce both immediate and poststimulation inhibition, whereas VRT stimulation only induces a poststimulation effect, with no direct effect on the nerves. The probable explanation for this poststimulation effect is that we used a normal animal model with intact neural pathways of micturition since we did not cut or injure the central or peripheral nerves. Thus, the electrical signal caused by VRT or DRT stimulation might start upstream in the spinal cord and brain and then descend to the bladder after being processed by the central nervous system. Previous studies have demonstrated that sacral neuromodulation activates the primary afferent nerves that project into the spinal cord and trigger the release of multiple neurotransmitters, such as GABA_A_, glycine, and opioid, which in turn modulate the micturition reflex [17, 18]. Further studies are needed to determine whether this is a result of a direct effect on efferent nerves or a poststimulation effect and the specific mechanisms.

To optimize treatment using electrical nerve stimulation, it is important to identify the superiority of the different VRTs and DRTs involved in micturition. In this study, we found that only stimulation of L6 VRT caused bladder contraction at the current parameters, indicating that L6 VRT is likely superior in producing detrusor muscle contraction. The mechanism is complex. It is well known that nerve fibers can be artificially stimulated by electrical current pulses delivered through electrodes located around the fibers. The direct outward flowing current with suprathreshold amplitude transmits along the fiber’s membrane or generates a suitable extracellular potential distribution in the vicinity of the fibers, resulting in membrane depolarization [19–21]. The threshold at which an action potential is generated depends on the fiber type and size and the stimulation parameters [22]. According to some studies on microsurgical anatomy of the lumbosacral VRTs, the VRT consists of several rootlets with similar calibers [23, 24]. Thus, because the stimulation parameters, fiber type, and caliber of the rootlets in the VRTs are consistent, we hypothesized that the number of rootlets involved may affect bladder contraction. The hypothesis was supported by our neuron retrograde tracing experiment.

Transneuronal transport of PRV is a promising technique for the identification of functional neural circuits. PRV can be picked up by nerve fibers at the injection site, undergo retrograde transport to the neuron soma, and then be transmitted along a chain of synaptically linked neurons [25]. An important feature of PRV compared to other neuroanatomic tracing methods, such as fluorescent dyes or horseradish peroxidase, is that it is transmitted from an infected neuron to other neurons only via their synaptic connections, thus precisely defining the specific pathways involved in neuron-to-neuron signaling [26]. The results in this study showed that PRV-positive neurons were found from L5 to S2 in the spinal cord and DRGs, mostly concentrated in the L6 SPN and the S1 DRG. In the nervous system, which controls the bladder, the SPN is where most sensory and motor nerves are centralized, whereas the DRG is where most sensory neurons are concentrated [27, 28]. From the density of PRV-positive neurons (L6 SPN > S1 SPN > L5 SPN > S2 SPN, S1 DRG > L6 DRG > S2 DRG > L5 DRG), it is reasonable to speculate that the L6 VRT contains the most motor nerve fibers involved in bladder control.

The results obtained from our experiments with DRTs showed that repeated S1 DRT stimulation produced longer BRC inhibition than L6 DRT stimulation, whereas the other DRTs had no effect on the BRC. This result is consistent with other studies. Ren et al. [7] used a rat model of BRC to determine the efficiency of L6 DRT stimulation and found that stimulation intensity was 50% lower with bilateral L6 DRT stimulation than unilateral stimulation, providing a similar BRC block. Zhang et al. [8] studied the inhibitory effect of sacral neuromodulation on reflex bladder activity and found that stimulation of S1 DRT was more effective than stimulation of other segments. The mechanism may be similar to that discussed earlier for VRT. The S1 DRT may contain more sensory nerve fibers involved in control of the bladder than other segments. Hence, the same parameters of stimulation induce more effective inhibition. Regarding the poststimulation inhibition, we assume that the inhibition of BRCs by DRT stimulation occurs primarily in the central nervous system by inhibition of the ascending or descending pathways of the spino-bulbo-spinal micturition reflex, which has been proposed in other studies [29–31].

Our study has some limitations. First, although the fixed parameters make it easy to compare the effects of different ventral and dorsal spinal roots, the results might not be 100% accurate because of the heterogeneity of rats. Second, our stimulation period was short and could only demonstrate acute effects. Because continuous stimulation is used in clinical sacral neuromodulation, further studies are needed to demonstrate the long-term effects of ventral and dorsal spinal nerve root stimulation and determine the best stimulation parameters. Third, because we applied the stimulations in order, poststimulation and cumulative effects might have influenced our results. Fourth, we used one hook electrode placed on the spinal root and another ground electrode placed in the tail. However, this ground electrode could have potentially stimulated the genital area and thus inhibited the bladder reflex activity. Thus, a bipolar hook electrode without the ground electrode is preferred for further studies. In addition, isovolumetric bladder contraction is a nonpathologic micturition reflex, and the ureters were not ligated in the rats, although basal endogenous urine production is low. Finally, all of the experiments were performed on anesthetized rats, and we found that the anesthetic had some effect on the elicitation of BRCs. Further experiments using conscious cystometry can target the mechanism by which neuromodulation acts and the rootlets through which messages pass.

In conclusion, we established a convenient rat model with parameter-fixed stimulation to eliminate the influence of varied stimulation parameters and animal models and identified that L6 VRT was most efficient in producing detrusor muscle contraction, whereas S1 DRT was superior in inhibiting BRC. These results may improve sacral neuromodulation therapy by optimizing the areas of stimulation. The animal model established in this study could also be used for further investigation of the superiority of rootlets involved in micturition or other functions such as erection.

## MATERIALS AND METHODS

The animal experiments were authorized according to the Guidance Suggestions for the Care and Use of Laboratory Animals (issued by the Ministry of Science and Technology of the People’s Republic of China) and were approved by the Ethics Committee of the Affiliated Sixth Hospital of Shanghai Jiao Tong University. Thirty female Sprague-Dawley rats, weighing 250 to 280 g, were purchased from the Laboratory Animal Service Center of Shanghai Jiao Tong University and were housed at a temperature of 20°C to 25°C and 50% ± 5% humidity with free access to food and water and an alternating 12-hour light/12-hour dark cycle.

### Animal preparation

Twenty rats (Group A), were anesthetized with urethane (1.2 g/kg, intraperitoneal injection), and the body temperature was maintained at 37°C to 38°C with a heating pad during the experimental procedures.

**Figure 4:**
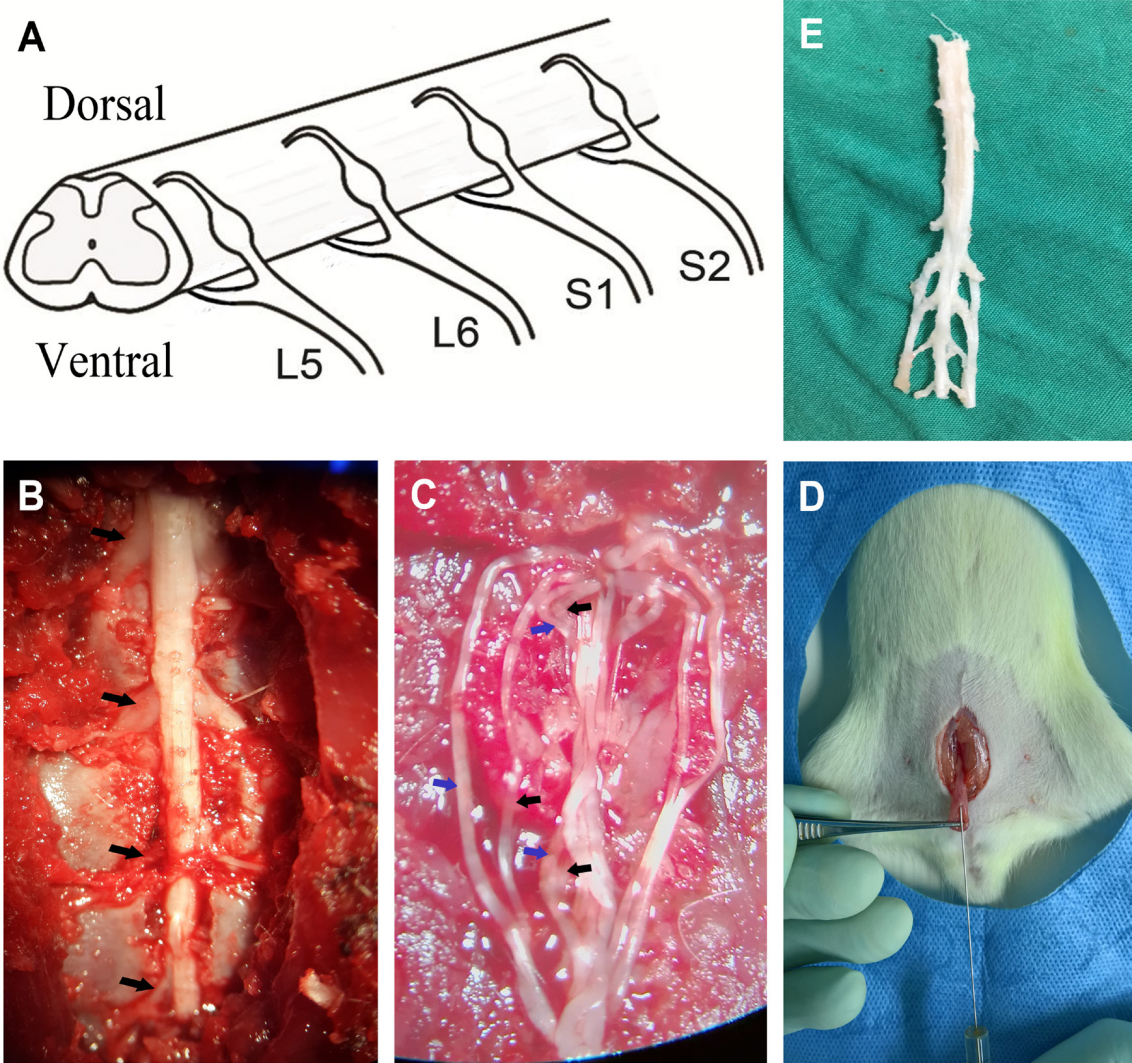
Images of the experiment process (**A**) Anatomic schematics showed the L5-S2 VRTs and DRTs. (**B**) Left L5-S2 spinal nerves were shown (black arrows). (**C**) The VRTs and DRTs were dissected (blue arrows: DRTs; black arrows: VRTs). (**D**) The bladder was exposed, and PRV was injected by a Hamilton syringe. (**E**) L5-S2 spinal segments and DRGs were removed for immunofluorescence experiment.

To record bladder contractions, a polyethylene catheter (PE-50) was inserted into the bladder via the urethra and secured with a suture tie. Glue was added around the urethral meatus to facilitate polyethylene catheter fixation and prevent urine leakage. The polyethylene catheter was connected to a syringe pump (WZ50CZ, China) and a pressure transducer (MLT0380/D; ADInstruments Pty Ltd, Sydney, Australia) via a T-stopcock. The intravesical pressure signal was stored by a biological signal collecting and processing system (PowerLab 4/26; ADInstruments). The animal was then placed in its prone position, and the skin on the surgical field was shaved. A dorsal midline incision was made, and laminectomy was performed to expose the spinal cord and nerves under a surgical microscope (Carl Zeiss, Oberkochen, Germany) (magnification 10×). Then the spinal dura was cut, and dorsal/ventral spinal roots from L5 to S2 on the left side were dissected (Figure 4B and 4C). Mineral oil highly resistant to electricity (35°C–37°C) was added to cover the surgical region for insulation and prevention of drying. A silver hook electrode was placed under each spinal root for stimulation, and a needle electrode under the skin of the tail served as the ground.

### Stimulation protocol

To induce BRCs, room temperature saline was infused into the bladder via the syringe pump at a rate of 50 μL/min. When the first micturition reflex (defined as a bladder contraction magnitude > 10 cm H_2_O) was evoked, the infusion rate was then lowered to 10 μL/min. After three to five consecutive contractions, isovolumetric rhythmic bladder contractions were established and saline infusion was then terminated.

To eliminate the variables of different stimulation parameters, monophasic square pulses (0.1-millisecond pulse width, 10-Hz frequency, 0.2-mA intensity, 30-second persistent period), according to previous studies [7, 32–35], were delivered via the hook electrode first to the DRTs in the order of L5, L6, S1, and S2 and then to the VRTs in the same order. Next, stimulation was performed until at least a normal BRC was seen to detect whether there was any poststimulation effect and to allow the bladder reflex to recover. The frequency/interval and amplitude of BRCs were evaluated.

### PRV retrograde tracing

Ten rats (Group B) were anesthetized with pentobarbital (50 mg/kg intraperitoneal), and an abdominal incision was made to expose the urinary bladder. Four microliters of PRV (PRV-152, 1 × 10^9^ pfu/mL, courtesy of Prof. Guangzhi Tong, Department of Swine Infectious Diseases, Shanghai Veterinary Research Institute, Chinese Academy of Agricultural Sciences), encoding for green fluorescent protein (GFP), were slowly injected into the ventral bladder detrusor muscle with a Hamilton syringe (Figure 4D). During injection, the needle remained *in situ* for 1 minute. Medical adhesive was used to prevent leakage of the virus when removed, and pressure was applied to the injection site. Then the area was thoroughly rinsed with saline, and the incision was closed. After being kept alive for 72 hours, animals were perfused transcardially with 0.1 M of phosphate-buffered saline, followed by cold 4% paraformaldehyde. Spinal cord and the DRGs from L5-S2 were removed (Figure 4E) to fixative for 4 hours and then transferred to a 30% sucrose solution for another 24 hours before cutting. Transverse sections (20 μm) taken from L5-S2 spinal cord segments and DRGs were processed on a cryostat microtome. A series of one in 10 sections were collected for observation and statistics. All PRV-positive cells displaying typical neuronal morphology in the SPN and DRG were counted separately at 100× magnification.

### Data analysis

All data are expressed as mean ± standard error of the mean and analyzed using one-way analysis of variance with the Tukey HSD test for post hoc analysis or the paired samples *t*-test, as appropriate. Statistical analysis was performed using SPSS 17.0 (SPSS, Chicago, IL). Statistical significance is defined as two-tailed *P* < 0.05.

## Abbreviations

BRC: bladder reflex contraction
DRG: dorsal root ganglions
DRT: dorsal root
PRV: pseudorabies virus
SPN: sacral parasympathetic nucleus
VRT: ventral root
GFP: green fluorescent protein
